# Toward a better understanding of social learning, social deciding, and other-regarding preferences

**DOI:** 10.3389/fnins.2014.00362

**Published:** 2014-11-06

**Authors:** Steve W. C. Chang, Masaki Isoda

**Affiliations:** ^1^Department of Psychology, Yale UniversityNew Haven, CT, USA; ^2^Department of Neurobiology, Yale University School of MedicineNew Haven, CT, USA; ^3^Department of Physiology, Kansai Medical University School of MedicineHirakata, Osaka, Japan

**Keywords:** social neuroscience, prosocial behavior, competitive behavior, oxytocin, social gaze orienting

What makes us do things like cooperate with others, perform altruistic behaviors, or be empathetic toward others? What neural circuits compute, and how hormones modulate, social behaviors? Our brains were evolved to function in complex social environments, demanding us to naturally tune our behaviors to social information (Wilson, 2000). Social behaviors often define who we are and give rise to individual identity as well as group identity. The papers appearing in the E-Book on *Neural basis of social learning, social deciding, and other-regarding preferences* cover a large part of current debate in, and expand our knowledge of, social behaviors from the perspectives of neural networks and neuromodulators involved, as well as unique behavioral strategies engaged. This collection addresses outstanding questions in the neurobiology of human and non-human animal social behaviors in the form of original research articles, reviews, and perspective-type papers.

Lee and Harris sets the stage by reviewing useful ways to effectively combine the knowledge gained from the tradition of social psychology research and more recently emerged research in neuroeconomics, in order to better understand social behaviors and their neural correlates (Lee and Harris, 2013). Taking a neuroethological viewpoint, Gariépy et al. review the similarities and differences in social learning across human and other species, and discuss the neural circuits involved in social learning (Gariépy et al., 2014). Focusing on the importance of economical computations involved in social behaviors, Hillman discusses the fundamental role of cost-benefit calculations for competitive social interactions (Hillman, 2013). Furthermore, emphasizing the significance of social context in shaping social behaviors, van den Bos et al., in an original research article, provide novel evidence that social identity is a strong driver of costly competitive behavior in humans engaged in a multi-player auction game (Van den Bos et al., 2013). The authors further show that the basal levels of testosterone could predict this competition-driven behavior, endorsing the notion that neuromodulators are crucial for context-dependent social processing.

Conceptualizing complex social behaviors in a computational framework is a challenging task. In a hypothesis and theory article, Chang proposes a coordinate transformation framework, a scaffold borrowed from the tradition of sensorimotor research (Andersen et al., 1993; Snyder, 2000), for characterizing how individual neurons encode social variables about self and others during social interactions (Chang, 2013). Furthermore, Zak and Barraza, in their review paper, propose a mathematical model for describing the phenomenon of collective action in order to help explain how empathy and other cognitive variables modulate altruistic behaviors in humans (Zak and Barraza, 2013).

Several papers in this issue argue for a specialized role of medial prefrontal/frontal cortical regions for guiding social behaviors. In an opinion piece, Lavin et al. propose that the human anterior cingulate cortex serves as a network hub in the brain for integrating the neural processes underlying social context processing, decision-making, and empathy (Lavin et al., 2013). In a perspective article, Apps et al. discuss a socially-specialized role of the gyrus portion of the human midcingulate cortex in predicting and monitoring decision outcomes when interacting with others (Apps et al., 2013). Furthermore, in a review paper, Isoda and Noritake deliberate why the dorsomedial frontal cortex is specialized for mediating theory of mind or mentalizing by discussing the key functions of this region in executive inhibition, self-other distinction, prediction under uncertainty, and perception of other's intention (Isoda and Noritake, 2013). A recent finding demonstrating the role of this brain region in shifting one's strategy in accordance with the strategy of a competitive opponent (Seo et al., 2014) strongly supports their prediction.

No one will argue that mother-infant interaction is one of the most critical social behaviors that influence behaviors that last one's entire lifespan. In an original research article, Strathearn and Kim report that the hemodynamic activations in the amygdala in mothers are strongly modulated by infant identity (own-infant vs. unknown-infant) and valence displayed (happy vs. sad faces) from the images of infants, indicating that positive or negative values associated with infant face are processed in strikingly different ways depending on social context (Strathearn and Kim, 2013). In another original research article, Ho et al. investigated dispositional empathy and stress sensitivity in mothers during a maternal decision-making scenario and found that different components of maternal dispositional empathy map onto distinct parts of the brain, spanning various subcortical and cortical regions (Ho et al., 2014).

Facial expression serves as a key source of social information in human and non-human primates. In a perspective article, Gothard reviews how distributed networks of sensory, motor, affective, as well as motivational systems coordinate to generate facial expression, focusing particularly on the pathways between the amygdala and the midcingulate areas for transforming emotion-related signals into facial motor signals (Gothard, 2014). It is well known that salient features in the environment capture one's attention (Itti and Koch, 2000). Social stimuli, such as faces and vocal calls, are particularly powerful at evoking orientation, probably reflecting their evolutionary importance for survival and reproduction. In an original research article, Dal Monte et al. show that exogenous oxytocin boosts social attention in rhesus macaques while viewing the faces of conspecifics (Dal Monte et al., 2014). The authors report that exogenous oxytocin increases gaze fixations to the eyes relative to the mouth, suggesting the role of oxytocin in actively distributing attentional resources toward the eye region when viewing faces. In another original research article on social attention, Ebitz et al. used a visual distractor task in rhesus macaques using social and non-social images to show that greater pupil constriction is observed when viewing social images, suggesting that pupillary responses involved in attention take social relevance into account (Ebitz et al., 2014). Using a novel scene-based search array task, Solyst and Buffalo report that rhesus macaques spend more time viewing conspecific images when the images contain socially salient features, such as those with direct gaze and redder sex skin (Solyst and Buffalo, 2014). The authors further demonstrate that this preferential viewing was not driven by lower-level saliency attributes in these images. Collectively, these articles illustrate how the brain prioritizes social information.

Recently, there has been much interest in testing how neurons sensitive to primary reinforcement and action monitoring respond to socially-rewarding events and socially-relevant actions (Izuma et al., 2008; Smith et al., 2010; Yoshida et al., 2011, 2012; Azzi et al., 2012; Báez-Mendoza et al., 2013a; Chang et al., 2013). In a review paper, Báez-Mendoza and Schultz discuss the role of the striatal neurons in selectively integrating rewards and actions across self and others, potentially mediating credit assignment between rewards and agency during social interactions (Báez-Mendoza and Schultz, 2013b). In an original research article, Kazama et al. show new causal evidence that neonatal lesion to the orbitofrontal cortex, implicated in value representation in rhesus macaques (Padoa-Schioppa and Assad, 2006; Kennerley et al., 2011), impairs the ability later in life in adjusting behavioral responses to changing reward values (Kazama et al., 2014).

The papers appearing in the E-Book collectively highlight new advances and emerging interests in the field of social neuroscience. The field is rapidly growing, providing many interesting insights into the neural underpinnings of social behavior. However, many challenges lie ahead. How does the brain know when to enhance social processing? What are some fundamental computational principles in the shared neural circuits across social and non-social behaviors? How do different parts of the brain or distinct subpopulations of neurons orchestrate social computation? These are just some of the interesting questions and challenges that remain before us.

## Author contributions

Steve W. C. Chang and Masaki Isoda wrote the current paper and served as the editors of this Research Topic E-Book.

### Conflict of interest statement

The authors declare that the research was conducted in the absence of any commercial or financial relationships that could be construed as a potential conflict of interest.
